# Post-treatment neutrophil-to-lymphocyte ratio in predicting prognosis in patients with metastatic clear cell renal cell carcinoma receiving sunitinib as first line therapy

**DOI:** 10.1186/2193-1801-3-243

**Published:** 2014-05-12

**Authors:** Yong Hyun Park, Ja Hyeon Ku, Cheol Kwak, Hyeon Hoe Kim

**Affiliations:** Department of Urology, The Catholic University of Korea, Seoul St. Mary’s Hospital, Seoul, Korea; Department of Urology, College of Medicine, Seoul National University, 101 Daehak-no, Jongno-gu, Seoul, 110-744 Korea

**Keywords:** Neutrophil-to-lymphocyte ratio, Renal cell carcinoma, Sunitinib, Response

## Abstract

**Purpose:**

Neutrophil-to-lymphocyte ratio (NLR) was evaluated as a prognostic factor in patients with metastatic clear cell renal cell carcinoma (RCC) receiving sunitinib as first line therapy.

**Methods:**

Between December 2005 and December 2011, 109 patients with metastatic clear cell RCC were treated with sunitinib. The values of NLR were assessed at two time points: at baseline (pre-treatment) and on day 1 of the second cycle (post-treatment). The prognostic significance of NLR on treatment outcome was evaluated with adjustment for known confounding risk factors.

**Results:**

The median follow-up duration after sunitinib treatment was 24 months. There was no association between the pre-treatment NLR and tumor response (median pre-treatment NLRs: 2.2 for partial response [PR], 2.3 for stable disease [SD], and 1.9 for progressive disease [PD]; p = 0.531). However, lower post-treatment NLR (1.1 for PR, 1.2 for SD, 2.3 for PD; p = 0.021) and larger reduction in NLR after treatment (-45.8% for PR, -45.6% for SD, 14.8% for PD; p = 0.009) was significantly associated with a better tumor response. When the patients were divided into two subgroups according to the cutoff value of the post-treatment NLR 1.1, the differences in median cancer-specific survival were observed between subgroups (not reached vs. 19.0 months, p = 0.012). In multivariate analysis, body mass index, MSKCC risk group, serum hemoglobin, and post-treatment NLR were significantly associated with cancer-specific mortality.

**Conclusions:**

Higher post-treatment NLR was associated with poor prognosis. An early reduction in the NLR after sunitinib treatment may indicate survival benefit in patients with metastatic clear cell RCC.

## Introduction

Inflammation plays a critical role in many aspects of cancer, including cancer development, growth, and progression (Mantovani et al. 2008). It is now established that progression of cancer is not only determined by the tumor characteristics but also by the host response. Several studies recently revealed that systemic inflammatory markers were associated with poorer prognosis in cancer patients (Vakkila and Lotze 2004; McMillan 2008). In patients with renal cell carcinoma (RCC), serum C-reactive protein (CRP) level (Steffens et al. 2012; Yasuda et al. 2012; Saito and Kihara 2010) and the Glasgow Prognostic Score (Ramsey et al. 2007; Lamb et al. 2012) have prognostic significance.

The relative difference in the neutrophil and lymphocyte counts, the neutrophil-to-lymphocyte ratio (NLR), has attracted the interests of investigators as an emerging systemic inflammatory marker. A high preoperative or pre-treatment NLR was identified as an independent prognostic factor associated with poor survival in various cancers, including breast cancer, colon cancer, gastric cancer, and meshothelioma (Azab et al. 2012; Chua et al. 2011; Kao et al. 2010; Cetin et al. 2013). Studies targeting patients with RCC found that an increased preoperative or pre-treatment NLR was associated with poor prognosis (Cetin et al. 2013; Ohno et al. 2010, 2012; Keizman et al. 2012). However, to the best of our knowledge, the association between the NLRs at different time points and treatment outcome in metastatic RCC patients receiving sunitinib has not been previously reported. In this study, we evaluated whether the NLR can be used as a surrogate indicator of treatment efficacy and prognosis in patients with metastatic clear cell RCC receiving sunitinib as first line therapy.

## Materials and methods

Between December 2005 and December 2011, 113 patients with histologically confirmed metastatic clear cell RCC were treated with sunitinib as first line therapy. We excluded four patients who did not undergo radical or partial nephrectomy for a primary lesion. Thus, 109 patients who underwent curative surgery for a primary lesion were included in this study: radical nephrectomy in 87 patients (79.8%) and partial nephrectomy in 22 (20.2%). The tumor stage was determined according to the 7th TNM classification of the Union Internationale Centre le Cancer (UICC) and the American Joint Committee on Cancer (AJCC) guidelines (Edge and Compton 2010) and grades of tumor were determined according to the Fuhrman’s grading system (Fuhrman et al. 1982). Sunitinib was administered orally at a dose of 50 mg once daily in 6-week cycles consisting of a 4-week on and 2-week off schedule (Motzer et al. 2007). For patients who developed toxicity, doses were reduced in single decrements of 12.5 mg to a minimum dose of 25 mg daily. After approval by the Institutional Review Board at Seoul National University Hospital, we reviewed the patient clinical data for gender, age, body mass index (BMI), ECOG performance status, pre-treatment laboratory findings, and treatment outcomes. The NLR was calculated by dividing the neutrophil count by the lymphocyte count. The values of NLR were assessed at two time points: just before the first cycle (pre-treatment) and on day 1 of the second cycle (post-treatment).

Tumor response was assessed every three cycles with computed tomography, based on the Response Evaluation Criteria In Solid Tumors (RECIST) criteria (Eisenhauer et al. 2009) using laboratory tests, chest X-ray, and abdominal CT scans. Cancer-specific death was attributed to patients with evidence of cancer progression before death by reviewing the patient’s medical records and/or the following codes using the International Classification of Diseases 10th revisions (ICD-10 code C64) from the database of the Korea National Statistical Office.

All statistical analysis was performed using IBM SPSS software, version 19.0 (SPSS, Inc., an IBM Company, Chicago, Illinois, USA). Data were summarized with the number of subjects and the mean or median value. Demographics and clinical parameters were analyzed with the chi-square test for categorical variables and with the Mann–Whitney *U* test or the Kruskal–Wallis test, as appropriate for continuous variables. A receiver operating characteristics (ROC) curve was constructed to estimate the optimal cut-off value of NLR. The cancer-specific survival rates were estimated using the Kaplan-Meier method and compared using the log rank test. A multivariate analysis was performed by Cox proportional hazard regression model for variables significant on univariate analysis. Two-sided null hypotheses of no difference were rejected if p-values were less than 0.05, or, equivalently, if the 95% confidence interval (CI) of hazard ratio estimates excluded 1.

## Results

### Baseline characteristics and treatment outcomes

Pre-treatment and post-treatment NLRs were available in all patients. Table 1 shows the baseline characteristics of the entire study population. The median follow-up duration after sunitinib treatment was 24 (interquartile range [IQR] 10–35) months. Objective response after 3 cycles of sunitinib treatment was complete response (CR) 0% (n = 0), partial response (PR) 45.0% (n = 49), stable disease (SD) 34.9% (n = 38), and progressive disease (PD) 20.2% (n = 22). The median cancer-specific survival for the entire group was 26.0 (95% CI 16.7 ~ 35.3) months.

**Table 1:** **Baseline patient characteristics**

Factors	Distribution
Age*	58.5, 61.0 (49.0 ~ 67.0)
Gender (%)	
Male	88 (80.7)
Female	21 (19.3)
BMI (kg/m^2^)*	23.3, 23.2 (21.4 ~ 25.1)
ECOG performance status (%)	
0	74 (67.9)
1	31 (28.4)
2	4 (3.7)
MSKCC risk group (%)	
Good	37 (33.9)
Intermediate	69 (63.3)
Poor	3 (2.8)
Synchronous metastasis (%)	56 (51.4)
Multiple organ metastasis (%)	52 (47.7)
Hemoglobin (g/dℓ)*	12.7, 12.8 (11.6 ~ 13.9)
Albumin (g/dℓ)*	4.1, 4.2 (3.9 ~ 4.4))
Corrected calcium (mg/dℓ)*	9.5, 9.3 (9.0 ~ 9.6)
Pre-treatment NLR*	2.8, 2.3 (1.5 ~ 3.1)
Post-treatment NLR*	1.4, 1.2 (0.8 ~ 1.7)

*Values are expressed as mean, median (interquartile range).

### Analysis for pre-treatment and post-treatment NLRs

**Figure 1:**
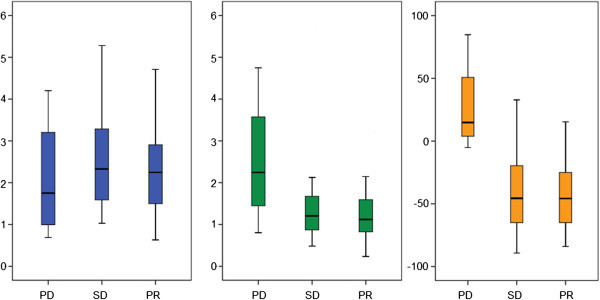
**Box plots showing distributions of NLR values according to the tumor response.** (1) Pre-treatment NLR. (2) Post-treatment NLR. (3) % Changes in post-treatment NLR.

The pre-treatment NLR ranged from 0.63 to 28.61, with a median value of 2.27 and a mean value of 2.82. There were no differences in the pre-treatment NLR by the patients’ characteristics, including age, gender, BMI, and ECOG performance status. There was no association between the pre-treatment NLR and the tumor response (median pre-treatment NLRs: 2.2 for PR, 2.3 for SD, and 1.9 for PD; p = 0.531) (Figure 1).

Across all study subjects, a 32.6% reduction in the median proportion of neutrophils (p < 0.001), a 5.6% increase in the median proportion of lymphocyte (p < 0.001), and a 38.3% reduction in the median post-treatment NLR occurred after first cycle of sunitinib treatment. The post-treatment NLR ranged from 0.24 to 5.42, with a median value of 1.18 and a mean value of 1.44. Better tumor response was significantly associated with the lower post-treatment NLR (1.1 for PR, 1.2 for SD, 2.3 for PD; p = 0.021) and the larger reduction in NLR after first cycle of sunitinib treatment (-45.8% for PR, -45.6% for SD, 14.8% for PD; p = 0.009) (Figure 1).

### Pre-treatment and post-treatment NLRs and survival outcomes

**Figure 2:**
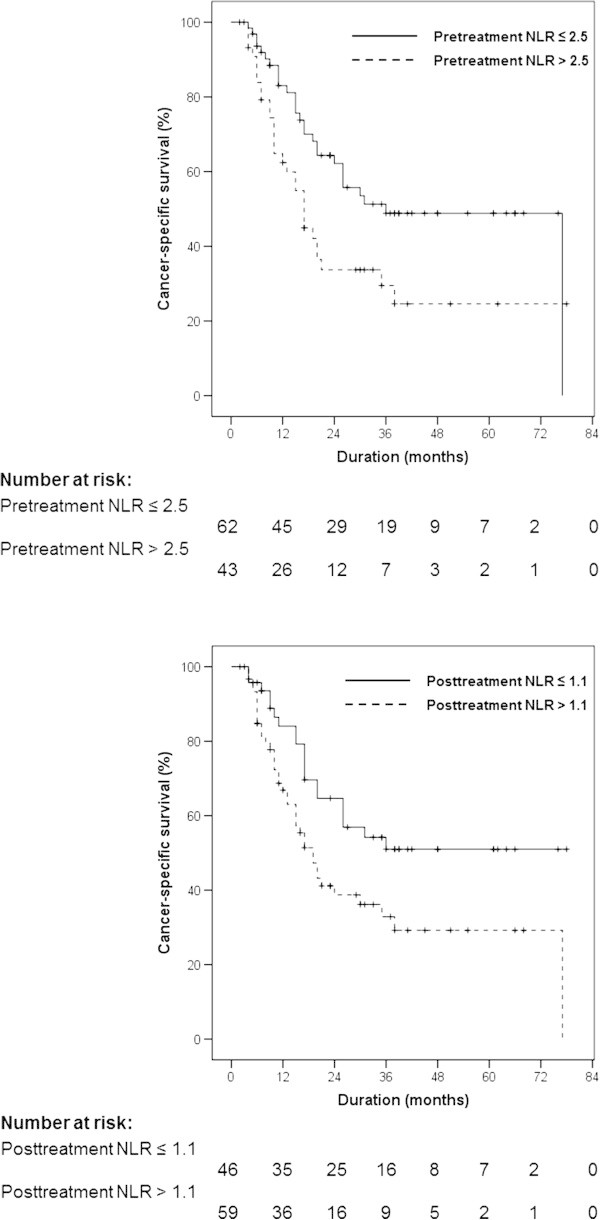
**Cancer-specific survival of the patients stratified by the pre-treatment and post-treatment NLR.** (1) By the cut-off point 2.5 of pre-treatment NLR. (2) By the cut-off point 1.1 of post-treatment NLR.

The patients were divided into two subgroups according to the cutoff value of the pre-treatment NLR 2.5, and post-treatment NLR 1.1 using receiver operating characteristics curve analysis. The differences in the cancer-specific survival according to the pre-treatment and post-treatment NLRs are presented in Figure 2. Kaplan–Meier univariate analysis showed significantly shorter median cancer-specific survival in patients with: (a) higher pre-treatment NLR (36.0 [95% CI 23.3 ~ 48.7] vs. 17.0 [95% CI 12.9 ~ 21.1] months, p = 0.014); and (b) higher post-treatment NLR (not reached vs. 19.0 [14.6 ~ 23.4] months, p = 0.012).On univariate analysis, BMI, MSKCC risk group, synchronous metastasis, multiple organ metastasis, serum hemoglobin, albumin, corrected calcium level, pre-treatment NLR, and post-treatment NLR were significantly associated with cancer-specific survival and multivariate analysis retained BMI, MSKCC risk group, serum hemoglobin level, and post-treatment NLR as independent predictive factors for cancer-specific survival (Table 2).

**Table 2:** **Prognostic factors associated with poor cancer-specific survival**

Factors	Univariate analysis			Multivariate analysis		
	HR	95% CI	***p-value***	HR	95% CI	***p-value***
Age (*cont.*)	1.004	0.980 ~ 1.028	0.762	-	-	-
Gender (%)						
Male	*Reference*			-	-	-
Female	1.087	0.574 ~ 2.059	0.797	-	-	-
BMI (kg/m^2^) (*cont.*)	0.891	0.812 ~ 0.979	0.016	0.884	0.668 ~ 0.924	0.031
ECOG performance status (%)						
0	*Reference*			-	-	-
1	1.576	0.907 ~ 2.807	0.105	-	-	-
2	1.598	0.879 ~ 5.131	0.457	-	-	-
MSKCC risk group (%)						
Good	*Reference*			*Reference*		
Intermediate	2.743	1.444 ~ 5.212	0.002	2.841	1.191 ~ 6.777	0.019
Poor	10.155	2.168 ~ 47.569	0.003	7.503	1.282 ~ 40.904	0.025
Synchronous metastasis (%)	1.855	1.093 ~ 3.146	0.022	1.598	0.783 ~ 3.260	0.197
Multiple organ metastasis (%)	1.769	1.033 ~ 3.029	0.038	1.237	0.624 ~ 1.736	0.789
Hemoglobin (g/dℓ) < reference value	0.672	0.565 ~ 0.800	0.001	0.756	0.595 ~ 0.959	0.021
Albumin ≥ 3.5 g/dℓ	0.442	0.262 ~ 0.744	0.002	1.000	0.493 ~ 2.028	1.000
Corrected calcium ≥ 10.0 mg/dℓ	1.352	1.090 ~ 1.677	0.006	1.132	0.876 ~ 1.462	0.345
Pre-treatment NLR ≤ 2.5	1.237	1.012 ~ 1.568	0.021	1.023	0.864 ~ 1.157	0.542
Post-treatment NLR ≤ 1.1	1.943	1.125 ~ 3.747	0.017	2.127	1.021 ~ 4.535	0.038

## Discussion

Prediction of tumor response is a critical issue in the treatment of metastatic cancer. However, only a few articles investigated the NLR as a potential prognostic factor in patients with metastatic RCC undergoing targeted therapy (Cetin et al. 2013; Keizman et al. 2012). The present study focused on the predictive value of the NLR in the outcome of patients with metastatic clear cell RCC receiving sunitinib as first line therapy. We found that a significant reduction in the NLR after sunitinib treatment, resulting from a reduction in neutrophils, and an increase in lymphocytes, is associated with a better tumor response and a longer cancer-specific survival in patients with metastatic clear cell RCC.

It has become clear that cancer progression is dependent on a complex interaction between the tumor and the host inflammatory response (Vakkila and Lotze 2004). Several lines of evidence demonstrated that RCC is related with inflammatory response. Wu et al. undertook a systematic meta-analysis to predict a role of systemic inflammatory response for survival in RCC (Wu et al. 2011). Three inflammatory markers were significantly associated with cancer-specific survival, with the pooled hazard ratio of 3.46 (95% CI 2.80 ~ 4.27) for elevated CRP, 3.22 (95% CI 2.25 ~ 4.62) for thrombocytosis, and 3.85 (95% CI 3.31 ~ 4.48) for elevated erythrocyte sedimentation rate (ESR). Therefore, strategies which reflect the host inflammatory response are promising prognostic factors.

Accumulated evidence has demonstrated that an elevated NLR represents a significant prognostic factor of poor clinical outcome in different types of cancer (Azab et al. 2012; Chua et al. 2011; Kao et al. 2010; Keizman et al. 2012). In addition to being measured easily, NLR is more valuable due to its special property as a combined factor of inflammation and host immune reaction. Ohno et al. first found NLR of value to predict recurrence in patients with non-metastatic RCC (Ohno et al. 2010). They reported that patients with NLR higher than 2.7 showed a significantly lower 5-year recurrence-free survival rates compared to those with NLR less than 2.7 (77.9 vs. 93.8%, p = 0.0205). They also investigated the clinical value of postoperative changes in NLR in patients with non-metastatic RCC; NLR significantly decreased after curative surgery, and increased at recurrence of RCC (Ohno et al. 2012). The magnitude of NLR changes after treatment may correlate with the bulk of disease. In our study, we can replicate this finding; changes in NLR after sunitinib treatment were correlated with the tumor response.

In patients treated with tyrosine kinase inhibitors for metastatic RCC, patients with higher pre-treatment NLR had poorer prognosis than those with lower pre-treatment NLR (Cetin et al. 2013; Keizman et al. 2012). These findings are consistent with our study. However, because the pre-treatment NLR might be highly correlated with the post-treatment NLR, we should clarify the roles of each NLR values in determining the prognosis in this study population. On multivariate analysis, the post-treatment NLR was a significant predictor of cancer-specific survival, thus, the strength of prediction seems to be greater in the post-treatment NLR that can also reflect the efficacy of treatment. We believe that our study has an advantage over prior studies due to the assessment of the post-treatment NLR as a new prognostic marker after sunitinib treatment.

It is unclear how elevated NLR would be responsible for treatment outcome. Some studies list possible mechanisms of NLR in cancer progression as follows: neutrophils, in response to IL-8 released by tumor cells, might contribute to cancer growth and metastasis by producing several cytokines (De Larco et al. 2004), and lymphocytes might be responsible for an inadequate immunologic reaction and, consequently, a weakened host defense against cancer (Hoffmann et al. 2002). Thus, the NLR may incorporate prognostic information of these two important parameters, and be a stronger prognostic factor of treatment outcome than either alone.

The current study has several limitations. First, although we tried to control possible factors which could influence the results by multivariate analysis properly, it is a retrospective study with a small number of patients in a single institution. Another potential limitation is that serum NLR was not compared to the extent of inflammatory cell infiltration within and surrounding the tumor. Such histological correlations should be considered in future analysis. However, despite these limitations, this study first established the post-treatment NLR as a surrogate indicator of treatment efficacy and prognosis in patients with metastatic clear cell RCC receiving sunitinib as first line therapy, which could help define the ideal patient population for aggressive follow-up and treatment.

## Conclusions

The present study shows that a significant reduction in the NLR after sunitinib treatment, resulting from a reduction in neutrophils, an increase in lymphocytes, is associated with a better tumor response and a longer cancer-specific survival in patients with metastatic clear cell RCC receiving sunitinib as first line therapy. Further studies are required to confirm a more detailed clinical relevance and biological mechanisms of post-treatment NLR.
